# Surface electromyogram, kinematic, and kinetic dataset of lower limb walking for movement intent recognition

**DOI:** 10.1038/s41597-023-02263-3

**Published:** 2023-06-06

**Authors:** Wenhao Wei, Fangning Tan, Hang Zhang, He Mao, Menglong Fu, Oluwarotimi Williams Samuel, Guanglin Li

**Affiliations:** 1 CAS Key Laboratory of Human-Machine Intelligence-Synergy Systems, Shenzhen Institute of Advanced Technology (SIAT), Chinese Academy of Sciences (CAS), and the SIAT Branch, Shenzhen Institute of Artificial Intelligence and Robotics for Society, Shenzhen, 518055 China; 2 Shenzhen College of Advanced Technology, University of Chinese Academy of Sciences, Shenzhen, Guangdong 518055 China; 3 School of Computing and Engineering, University of Derby, Derby, DE22 3AW UK; 4 Data Science Research Center, University of Derby, Derby, DE22 3AW UK

**Keywords:** Sensorimotor processing, Neuronal physiology

## Abstract

Surface electromyogram (sEMG) offers a rich set of motor information for decoding limb motion intention that serves as a control input to Intelligent human-machine synergy systems (IHMSS). Despite growing interest in IHMSS, the current publicly available datasets are limited and can hardly meet the growing demands of researchers. This study presents a novel lower limb motion dataset (designated as SIAT-LLMD), comprising sEMG, kinematic, and kinetic data with corresponding labels acquired from 40 healthy humans during 16 movements. The kinematic and kinetic data were collected using a motion capture system and six-dimensional force platforms and processed using OpenSim software. The sEMG data were recorded using nine wireless sensors placed on the subjects’ thigh and calf muscles on the left limb. Besides, SIAT-LLMD provides labels to classify the different movements and different gait phases. Analysis of the dataset verified the synchronization and reproducibility, and codes for effective data processing are provided. The proposed dataset can serve as a new resource for exploring novel algorithms and models for characterizing lower limb movements.

## Background & Summary

Intelligent human-machine synergy systems (such as rehabilitation equipment^1^, active prostheses^2–5^, and exoskeletons^6^) have developed rapidly with the continuous advancement in the medical, robotics, and computing fields. A key driver of these systems is the surface electromyogram (sEMG) signals acquired non-invasively that can be observed prior to the initiation of certain muscle-driven movements^7^. Thus, sEMG has been widely used in a broad spectrum of biomedical and non-biomedical applications that require the prediction of human motion intention^8^. For upper limb movement intent characterization, the Ninapro repository^9,10^ (a publicly accessible database) provides a considerable amount of datasets, including sEMG, joint angle (kinematics), and force (kinetics) data with corresponding labels to support the research community. Similarly, there are a few publicly available sEMG datasets for lower limb movement characterization, which have helped advance research and development to an extent in the field of rehabilitation robotics and the likes^11–17^. However, these lower limb sEMG datasets are limited in a number of ways that preclude their wide usage by researchers in the field. For instance, the currently available lower limb datasets do not directly provide detailed gait phase labels, which are essential for conducting appropriate investigations on gait phase characterization. Although gait phases can be computed from a few of the available datasets^15–17^ that include heel strike and toe-off information, this typically requires complex processing steps. At the same it is difficult to ensure fairness in the comparison of the accuracy of some machine learning algorithms due to the non-directly available labels for the gait phases. In addition, some of the existing lower limb datasets only considered recording data while subjects walk at various speeds on stairs or ground terrains^11–13^, without involving discrete movements that are needed in rehabilitation research and development^18,19^. Based on these reasons and the limitation of the existing publicly available lower limb datasets highlighted in Table 1, there is a need to provide a more comprehensive public lower limb datasets that could advance research, development, and innovation in the related domains. Table 1 presents the research demand versus characteristics of the available datasets, highlighting the drawbacks of the existing datasets, which the current study seeks to address.

**Table 1:** Research demand versus characteristics of the available datasets for lower limb movement characterization.

Existing Related Works		EMG Signals	Joint Angle	Joint Torque	Movement Label	Gait Phase Label	Body Data
Algorithm or Analyse	Movement classification Reference^37–43^	In need	Not necessary	Not necessary	More than 16 types	Not necessary	Not necessary
	Gait phase classification References^44–47^	In need	Not necessary	Not necessary	Not necessary	In need	Not necessary
	Joint angle prediction References^44,48–51^	In need	In need	Not necessary	Not necessary	Not necessary	Not necessary
	Torque prediction References^52,53^	In need	Not necessary	In need	Not necessary	Not necessary	Not necessary
	Individual differences References^54^	In need	In need	In need	Not necessary	Not necessary	In need
Public Datasets	HAR-SEMG dataset^14^	Included	Not available	Not available	Including five types	Not available	Incomplete
	L. Moreira dataset^13^	Included	Included	Included	Including seven types	Indirectly available	Included
	C. Schreiber & F. Moissenet dataset^11^	Included	Indirectly available	Indirectly available	Including five types	Indirectly available	Included
	T. Lencioni dataset^12^	Included	Included	Included	Including five types	Indirectly available	Included
	ENABL3S dataset^15^	Include	Include	Not available	Including seven types	Include	Incomplete
	J. Camargo dataset^16^	Include	Include	Include	Including four types (multiple speeds in each type)	Include	Incomplete
	HuMoD database^17^	Include	Include	Include	Including eight types (multiple speeds in some types)	Include	Include
	E. Reznuck dataset^28^	Not available	Include	Partly include	Including eight types (multiple setup in some types)	Indirectly available	Include
	WBD dataset^29^	Not available	Include	Include	Including two types (multiple speeds in each type)	Indirectly available	Include
	RBD dataset^55^	Not available	Include	Include	Including one type (three speeds)	Indirectly available	Include

Note: The first row of Table 1 is a table head that lists the common data types in the lower limb movement. Rows 2 to 6 of Table 1 present five kinds of research work on lower limb movement and the scientific data used in each. Row 7 to 16 shows the public dataset of lower limbs and the data types provided in each.

To address the limitations of the available datasets, we designed an experiment that allowed the simultaneous acquisition of nine channels of sEMG, joint angle (kinematics), joint torque (kinetics) along with their corresponding labels from 40 healthy subjects who performed 16 different lower limb movements, and the obtained dataset is named as Shenzhen Institute of Advanced Technology Lower Limb Motion Dataset (SIAT-LLMD)^20^. In addition, codes that allow reading the data, pre-processing of sEMG, splitting of sEMG into analysis windows of various sizes, extraction of feature sets, normalization of extracted features, generation of sample data, and making log files are provided for complete handling of the data. In summary, this work provides a dataset (SIAT-LLMD)^20^ with additional types of movements and unified labels to promote the advancement of scientific research and comparison of the related algorithms in the field of lower limb movement characterization.

## Methods

### Participants

In this study, a total of 40 healthy adult subjects including 30 males and 10 females were recruited for the collection of the sEMG, kinematics, and kinetics data associated with multiple classes of lower limb movements. In order to protect the identity information of the subjects, we coded their names as Sub01 – Sub40. The average age across subjects is 24.5 years old (with a minimum and maximum age of 19 and 33, respectively); the average weight of the subjects is 63.8 Kg (with a minimum and maximum weight of 46.3 Kg and 85 Kg, respectively); the average height of the subjects is 1693 mm (with a minimum and maximum height of 1550 mm and 1820 mm, respectively); the average thigh length of the subjects is 391.4 mm (with a minimum and maximum thigh length of 325 mm and 455 mm, respectively); the average calf length of the subjects is 409.1 mm (with a minimum and maximum calf length of 355 mm and 477.5 mm, respectively); and the average foot length of the subjects is 224.2 mm (from the heel to the toe’s first phalangeal joint, with a minimum and maximum foot length of 200 mm and 255 mm, respectively). Meanwhile, a detailed description of each subject’s characteristics is presented in Table 2.

**Table 2:** Basic of information of the 40 recruited subjects.

Subject	Sex	Age (Years)	Weight (Kg)	Height (mm)	Thigh length (mm)	Calf length (mm)	Foot length (mm)
Sub01	male	22	68.7	1750	432.5	425.0	247.5
Sub02	male	24	61.3	1735	415.0	412.5	235.0
Sub03	male	25	66.6	1775	385.0	435.0	230.0
Sub04	male	27	59.3	1625	325.0	387.5	215.0
Sub05	male	24	64.0	1735	392.5	440.0	230.0
Sub06	male	23	68.0	1735	410.0	430.0	230.0
Sub07	male	24	81.0	1685	390.0	405.0	230.0
Sub08	male	24	49.0	1710	365.0	407.5	220.0
Sub09	male	25	64.8	1655	370.0	397.5	217.5
Sub10	male	33	56.5	1630	345.0	412.5	222.5
Sub11	male	26	57.0	1625	385.0	390.0	230.0
Sub12	male	23	64.5	1685	372.5	410.0	212.5
Sub13	male	23	72.0	1750	407.5	420.0	220.0
Sub14	male	25	70.0	1700	400.0	402.5	210.0
Sub15	male	24	83.0	1700	342.5	432.5	225.0
Sub16	male	24	61.0	1760	455.0	410.0	217.5
Sub17	male	25	82.0	1790	407.5	420.0	245.0
Sub18	male	25	85.0	1790	425.0	445.0	237.5
Sub19	male	24	62.6	1660	357.5	385.0	210.0
Sub20	male	23	78.4	1760	420.0	420.0	235.0
Sub21	male	23	50.3	1630	410.0	397.5	222.5
Sub22	male	27	71.8	1745	415.0	430.0	245.0
Sub23	male	23	65.6	1750	427.5	415.0	225.0
Sub24	male	28	73.8	1695	405.0	390.0	225.0
Sub25	male	27	62.3	1630	350.0	380.0	220.0
Sub26	male	25	64.4	1720	415.0	445.0	255.0
Sub27	male	28	82.6	1740	440.0	405.0	245.0
Sub28	male	25	54.5	1720	387.5	417.5	220.0
Sub29	male	25	63.3	1820	405.0	425.0	230.0
Sub30	male	25	55.0	1745	455.0	447.5	247.5
Sub31	female	25	47.3	1615	385.0	385.0	212.5
Sub32	female	22	56.6	1675	395.0	422.5	215.0
Sub33	female	24	46.3	1560	357.5	372.5	205.0
Sub34	female	23	50.0	1660	402.5	395.0	220.0
Sub35	female	22	52.1	1630	385.0	385.0	205.0
Sub36	female	24	60.5	1630	375.0	390.0	210.0
Sub37	female	25	61.3	1630	345.0	405.0	210.0
Sub38	female	24	57.0	1550	350.0	355.0	200.0
Sub39	female	19	54.0	1620	372.5	397.5	215.0
Sub40	female	22	67.1	1700	375.0	417.5	220.0

The participants were recruited from the Shenzhen Institute of Advanced Technology, Chinese Academy of Sciences (SIAT-CAS) and the Shenzhen University via a publicity made on a social media application (specifically, the WeChat platform), describing the goal, experimental setup, and requirements of the study. Interested participants that signed up to take part in the study were given further descriptions (using photos and videos, etc.) about the goal and experimental procedures to ensure that they fully understood before participating in the study. Also, the participants were given the chance to ask related questions and responses were administered accordingly to ensure adequate understanding before the data collection began. The experimental design and protocol were reviewed and approved by the ethics committee led by the Institutional Review Board (IRB) of SIAT-CAS with an approval number of *SIAT-IRB-210315-H0555*.

### Experimental setup and Equipment

**Fig. 1:**
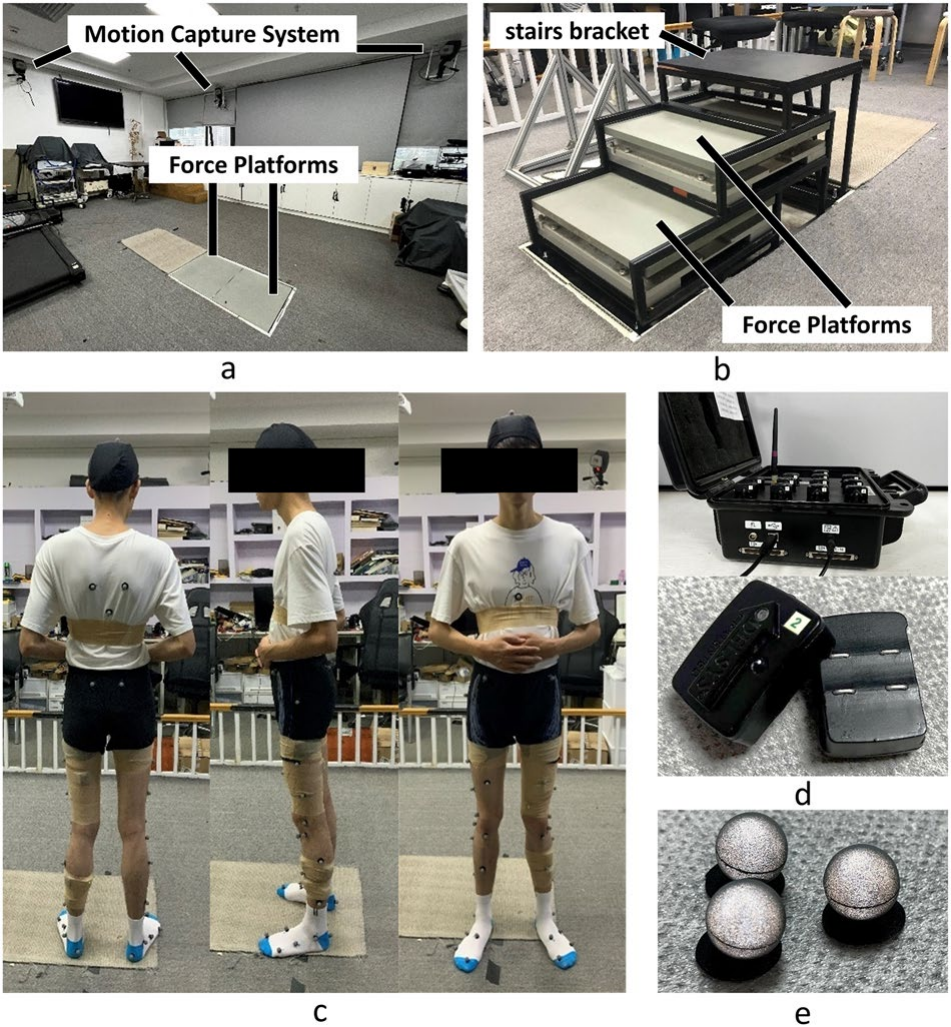
The experimental setup and equipment utilized. (**a**) The motion capture system and force platforms on the ground; (**b**) The force platforms on the stairs bracket; (**c**) A subject prepared for the experiment; (**d**) The sEMG DELSYS wireless acquisition system; (**e**) The cutaneous reflective markers.

A motion capture system with six cameras (Eagle, Motion Analysis, USA) was used to collect the original kinematic data; two six-dimensional force platforms (OR6-7, AMTI, USA) were used to record the ground reaction force (GRF); and a wireless sEMG acquisition system (DELSYS, USA) employed for the recording of the sEMG data (Fig. 1).

**Fig. 2:**
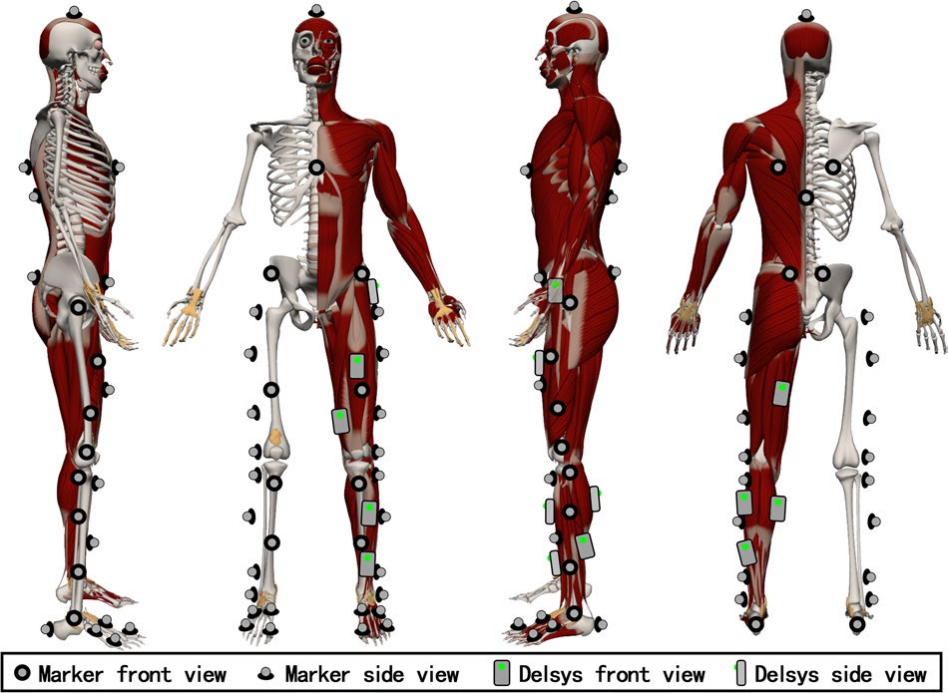
A representation of the Delsys sensors (for acquisition of sEMG signals) and the reflective markers (that aid capturing of kinematics data) placement utilized in the study.

The motion capture system was set at a sampling frequency of 60 Hz to track the three-dimensional (3D) trajectories of a set of configured 41 cutaneous reflective markers as shown in Fig. 2. This set of markers was used in the ‘Gait 2392’ OpenSim (a software from the National Center for Simulation in Rehabilitation Research) model^21^. The design of the markers is referred to in the guide for the usage of the Motion Capture and Analysis System and the recommendations of the International Society of Biomechanics^22,23^. The key markers in the bones used for the inverse kinematics calculation were placed based on guide from two skilled experimenters using the anatomical palpation method. The other markers used as redundancy for calculating missed markers were placed by the experimenters based on their personal experience. These redundant markers have zero weight during the calculation process. At the same time, to ensure the accuracy of the markers’ position, the subjects were asked to wear a light T-shirt, tight-fitting experimental shorts, take off their shoes, wear an experimental cap, and tuck their shirt into the shorts. Then, the T-shirts were wrapped with self-adhesive tensioners, and the subjects were instructed not to adjust the clothing midway into the experiment to ensure conformity with the protocol.

The ground reaction force (GRF) was recorded by the force platforms of 508 mm length and 464 mm breadth sampled at 1920 Hz, placed at the same height as the ground except when the subjects went upstairs or downstairs. For the stairs scenes, a stairs bracket that enables the force platforms to be set up in a desired manner (as shown in Fig. 1b) was designed. After setting up the platform, the height, width, and length of the stair are 150 mm, 260 mm, and 470 mm, respectively.

Thereafter, a total of nine-channel sEMG signals were collected by using a wireless acquisition system sampled at 1926 Hz, and the electrodes were firmly fixed on the tensor fascia lata, rectus femoris, vastus medialis, semimembranosus, upper tibialis anterior, lower tibialis anterior, lateral gastrocnemius, medial gastrocnemius, and soleus muscles on the left leg with a double-sided tape. During the experiment, we ensured that suggestions provided by the Institute of Neurology, Department of Clinical Neurophysiology were adhered to^24^. Before the placement of electrodes, the skin surfaces of the subjects were cleaned with alcohol cotton pads containing 75% alcohol to remove skin oil to avoid electrode impedance issues that may affect the quality of the recorded signals during the experiment. In addition, to avoid highly noisy signals that may result from vibration during the experiments, self-adhesive medical bandages were used to wrap and reinforce the EMG electrodes.

The whole system can collect the 3D trajectories of the marker set, GRF, and sEMG data, based on which, the kinematic and kinetic data can be calculated. Experimental data were collected after wearing the equipment (Fig. 1c). To ensure that the data is recorded appropriately, the reflective markers and sEMG sensors were carefully checked after each movement was performed by the subjects.

### Experimental protocol

**Fig. 3:**
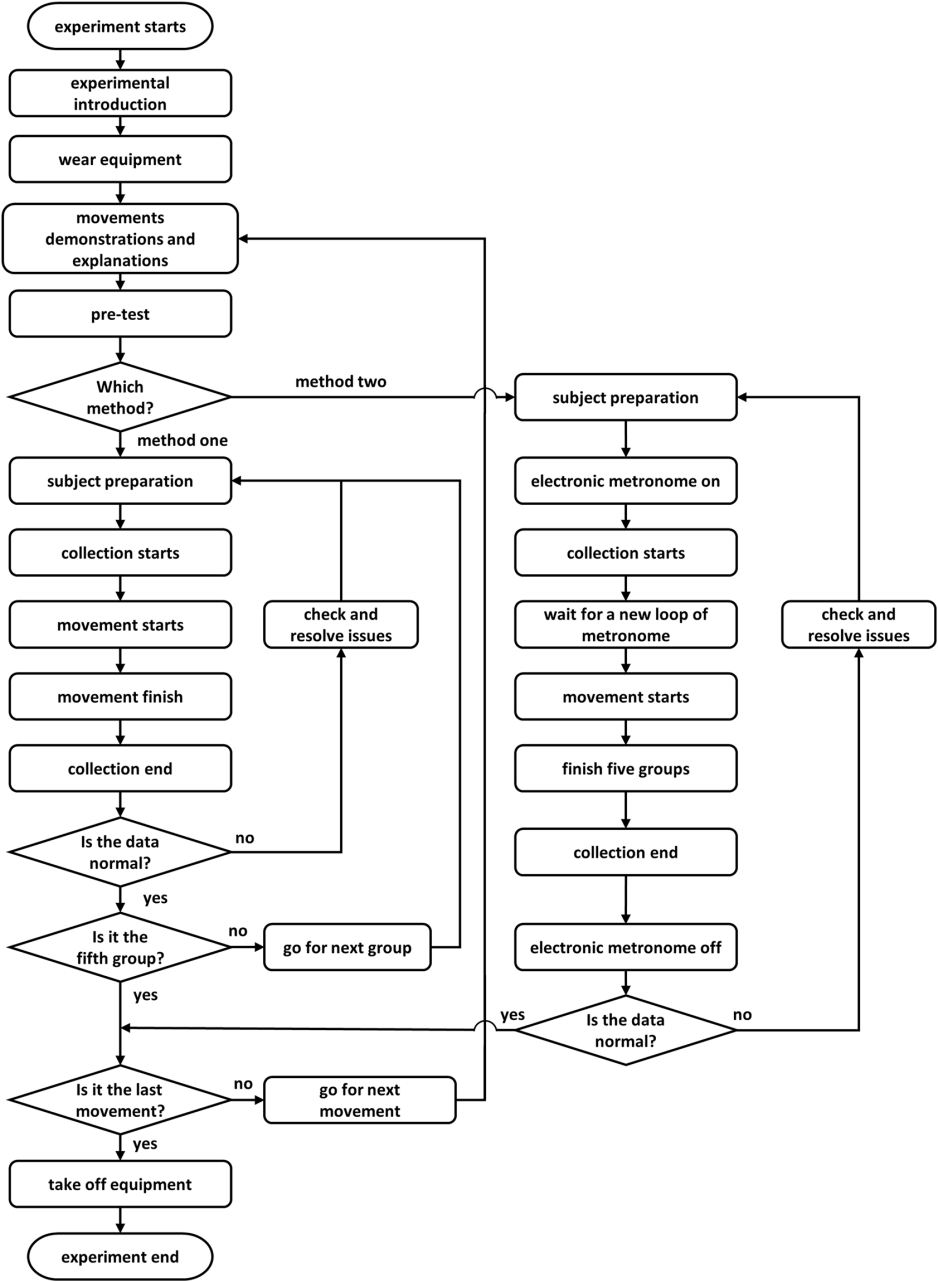
A flowchart representing the experimental procedure for data collection.

A total of 16 different lower limb movement tasks (including 12 different movements, no movement also known as the static state, and 3 different gaits) were considered in the data acquisition process. Precisely, these tasks include static (standing upright without making any movement), walking on a level ground, standing up, sitting down, walking upstairs, walking downstairs, knee lift, tipping the toe, leg lift forward, leg lift backward, knee lift then calf lift forward, leg lift sideward, heel strike, toe-off, lunging forward, and lunging backward (See the attached video and Table 3 for a clearer understanding of the experimental procedure for the tasks performed). According to the discrete nature of the movements, two methods (denoted as, method-1 and method-2) were employed for the data collection process which are represented in a flowchart shown in Fig. 3. In method-1, each participant is asked to perform a specific movement, and then repeat that same task nine times for gait phase related tasks and four times for the other tasks. In other words, each repetition constitute a raw data file, thus yielding a total of ten or five different raw data files per movement and subject. In contrast, for method-2, each participant is expected to perform a specific movement task five consecutive times in a row, yielding a single raw data file for the five trials. It is worth noting that method-1 was employed in collecting the data for static, walking on level ground, standing up, sitting down, stairs ascent, and stairs descent tasks because these tasks are designed to begin and terminate at certain intervals considering the experimental environment. Also, in method-2, the participants were required to follow an electronic metronome (1 second a beat, 8 seconds a loop) to complete each group of movements in 5 seconds and followed by 3 seconds of rest.

**Table 3:** Description of the 16 classes of lower limb movements considered in the study.

Movement	Abbreviation	Description
static	STC	The subject stands on the force platforms (playing the left and right foot each on the force platforms) with his toes pointing forward, both feet are about 100 mm apart, and hands placed on the abdomen.
walking	WAK	The subject stands about 2000 mm away from the force platforms with the hands placed on the abdomen. After prompted to begin the experiment, the subject walks through the platforms at a comfortable speed. In total, the WAK task is performed 10 times. And in the first five trials, the subject adjust the starting point to ensure that the left foot makes contact with the second force platform and the right foot makes contact with the first force platform. Meanwhile, the subject adjust the starting point to ensure that the right foot makes contact with the second force platform and the left foot makes contact with the first force platform.
stand up	STDUP	The subject sits on the chair (placed near the force platforms) with the back up straight, hands placed on the abdomen, and feet on the platforms (left foot on platform one and right foot on platform two) while maintaining a state. The subjects were asked to keep their lower limbs relaxed while sitting. Three seconds after the experiment begins, the subject stands up at a comfortable speed and maintains static posture for about 5 seconds during which the data was recorded.
sit down	SITDN	The subject stands naturally on the force platforms with the left foot placed on force platform one, right foot placed on force platform two, and hands placed on the abdomen. Three seconds after the experiment begins, the subject sits down on a chair placed near the force platforms at a comfortable speed and maintained the sitting position for about 5 seconds during which the data was recorded. The subjects were asked to keep their lower limbs relaxed while sitting.
upstairs	UPS	The subject stands on the ground directly in front of the staircase with hands placed on abdomen in a relaxed manner. Three seconds after the experiment begins, the subject walks up the stairs at a comfortable speed in the first five trials initiating the movement with the left foot. In the later five trials, the subject walks up the stairs in a similar but this time the movement is initiated with the right foot. Meanwhile, the subject maintains a static posture for 5 seconds after arriving at the top of the stairs.
downstairs	DNS	The subject stands at the top of the staircase with hands placed on the abdomen in a relaxed manner. Three seconds after the experiment begins, the subject walks down the stairs at a comfortable speed in the first five trials initiating the movement with the right foot. In the later five trials, the subject walks down the stairs in a similar but this time the movement is initiated with the left foot. Meanwhile, the subject maintains a static posture for 5 seconds after arriving at the ground (stairs base).
knee lift	KLFT	The subject stands at the force platforms (with the left foot on platform one and the right foot on platform two) with hands on the abdomen in a relaxed manner. When the movement begins, the subject flexes the left hip (sagittal plane), keeps the knee relaxed, and then puts down the leg.
tip the toe	TPTO	The subject stands at the force platforms (with the left foot on platform one and the right foot on platform two) with hands on the abdomen in relaxed manner. When the movement starts, the subject flexes the left hip (sagittal plane) to about 30° and keeps the leg straight. Then, the subject flexes the ankle, holds on for one second, relaxes the ankle, and puts down the leg.
leg lift forward	LLF	The subject stands at the force platforms (with the left foot on platform one and the right foot on platform two) with the hands on the abdomen in a relaxed manner. When the movement starts, the subject flexes the left hip (sagittal plane) to about 45°, keeps the leg straight, and then puts down the leg.
leg lift backward	LLB	The subject stands at the force platforms (with the left foot on platform one and the right foot on platform two) with the hands on the abdomen in a relaxed manner. When the movement starts, the subject flexes the left knee joint to about 80°, and then, puts down the leg.
leg lift sideward	LLS	The subject stands at the force platforms (with the left foot on platform one and the right foot on platform two) with the hands on the abdomen in a relaxed manner. When the movement starts, the subject flexes the left hip (coronal plane) to about 45°, keeps the leg straight, and then puts down the leg.
Knee lift then calf lift forward	KLCL	The subject stands at the force platforms (with the left foot on platform one and the right foot on platform two) with the hands on the abdomen in a relaxed manner. When the movement starts, the subject flexes the left hip (sagittal plane), keeps the knee relaxing, then extends the left knee joint until the leg becomes straight, and puts down the leg at last.
heel strike	HS	The subject stands at the force platform two facing force platform one with the hands on the abdomen in a relaxed manner. When the movement starts, the subject stretches out the left leg (initiating the movement) similar to level-ground walking. Then, the left heel strikes the force platform one, the left toe contacts the platform one and holds on. Finally, the left leg is retracted.
toe-off	TO	The subject stands at the force platform one facing force platform two with the hands on the abdomen in a relaxed manner. When the movement starts, the subject stretches out the right leg (initiating the movement) similar to level-ground walking. Then, the right heel strikes the force platform one, the right toe contacts the platform and holds on. Finally, the right leg is retracted.
lunge forward	LUGF	The subject stands at the force platform two facing force platform one with the hands on the abdomen in a relaxed manner. When the movement starts, the subject steps forward with the left leg and makes a lunge. Finally, the left leg is retracted.
lunge backward	LUGB	The subject stands at the force platform one facing force platform two with the hands on the abdomen in a relaxed manner. When the movement starts, the subject steps forward with the right leg and makes a lunge. Finally, the right leg is retracted.

The summary for the justification on why we selected the movements considered in our proposed dataset is given as follows:

Goal one: The movements in SIAT-LLMD should include the main functions of the lower limb motion during daily life activities such as WAK, UPS, DNS, STDUP, and SITDN. In addition, in cases where an individual needs to make a sidestep in a certain direction, then LLF, LLB, and LLS movements will be needed.

Goal two: The SIAT-LLMD should be able to offer support for research involving a single gait phase in a gait cycle. For instance, when an individual starts walking from standing, the movement of the front leg corresponds to the HS, and the back leg corresponds to the TO. The KLCL is designed to support taking longer strides. At the same time, the longer strides require lunge to heel strike while crossing a ditch, so we considered the LUGF and LUGB movements in our dataset. In addition, the calf is often lifted when climbing up stairs which led us to include KLFT movement.

Goal three: It is also important to include movements associated with the angle range of motion of the three joints (hip, knee, and ankle) as much as possible to comprehensively support the research of using sEMG to track joint motion. To achieve this goal, we chose the KLFT, LLB, and LLS to cover the angle range of the hip, KLFT to cover the angle range of the knee, TPTO for the angle joint, and lunge movement (LUGF and LUGB) for the angle with stretch.

The above-described experimental protocol was carried out after the equipment was successfully set up. And the entire experiment lasted for 90 to 140 minutes per subject, depending on the learning rate and required rest time of each subject while about 40 minutes was spent wearing the markers and delsys sensors. In addition, subjects were permitted to stop the experiment and take adequate rest whenever they felt fatigued.

### Data processing

The required Motion capture files can be obtained from the process mentioned in the previous section. Afterward, the following data processing procedures were performed: markers data processing, data extraction, kinematics and kinetics data calculation, and data alignment.

1. Markers data processing: The missing markers were fixed and named in accordance with the guidance presented in Fig. 2. These processes were carried out using the Cortex Software (Motion Analysis Company, USA). The function of “Rigid Body Join”, which ensures the correctness of the data by using the correct markers to fix the missing markers belonging to the same rigid, was preferred while fixing the missing markers. In addition, the smooth function was used on some frames following the suggestions from the Cortex Software, and a ‘.c3d’ file was finally generated as the output.
2. Data extraction: From the obtained ‘.c3d’ file, the sEMG data, ‘.trc’ file, and ‘.mot’ files were individually extracted. Meanwhile, the ‘.trc’ file and ‘.mot’ files were then used in the OpenSim software for calculating the corresponding kinematics and kinetics information.
3. Kinematics and Kinetics computation: Firstly, the STC and body weight data were used to scale the standard model for each subject. Secondly, the scaled model and the ‘.trc’ file were used to calculate the kinematics data associated with the joints. Thirdly, the ‘.mot’ file and kinematics data were used to calculate the kinetics associated with the joints, and the GRF data while the filter was set to 15 Hz.
4. Data alignment: Firstly, the kinematics and kinetics datasets were smoothed by the ‘smoothdata’ function (method: ‘sgolay’ window: 4) in Matlab. Secondly, the EMG data was used as a reference to upsample the kinematic and kinetic datasets. Finally, the sEMG, kinematics, and kinetic datasets were combined into a ‘.csv’ file and designated as the final data file.
5. Data trim and merge: For each of the STDUP and SITDN, the five data files were sequentially concatenated into one data file to keep the storage format consistent. Data lacking full GRF information were trimmed for the movements UPS, and DNS. The ten data files were concatenated into one data file for each WAK, UPS, and DNS, with the lift-foot-first group in the front and the right-foot-first group in the back.

Although sEMG signals usually require filtering before use, some studies still expect the direct application of raw data. So, raw EMG data and a set of codes have been provided in this work to enable easy exploration of our dataset by interested researchers. The codes provide different filters, normalization methods, and window settings that are suitable for the dataset, by virtue of which, researchers can easily obtain data that could be used for various analyses when considering statistical, machine learning, and other methods. It should be noted that the data lacking full GRF information in WAK is marked as NAN (Not a Number), and the NAN data needs to be excluded before using the enclosed codes to automatically process it. Besides, interested researchers may also use the data without excluding the NAN aspect but would need to either modify our codes or use a custom built codes.

### Label

This data set provides two types of labels. The first type is suitable for STC, STDUP, SITDN, KLFT, TPTO, LLF, LLB, LLS, KLCL, HS, TO, LUGF, LUGB, while the second type is suitable for WAK, UPS, and DNS. In addition, the code in this work could be used to extract samples with the required labels or one-hot labels for researchers expecting to do pattern recognition for specific movement classification.

**Fig. 4:**
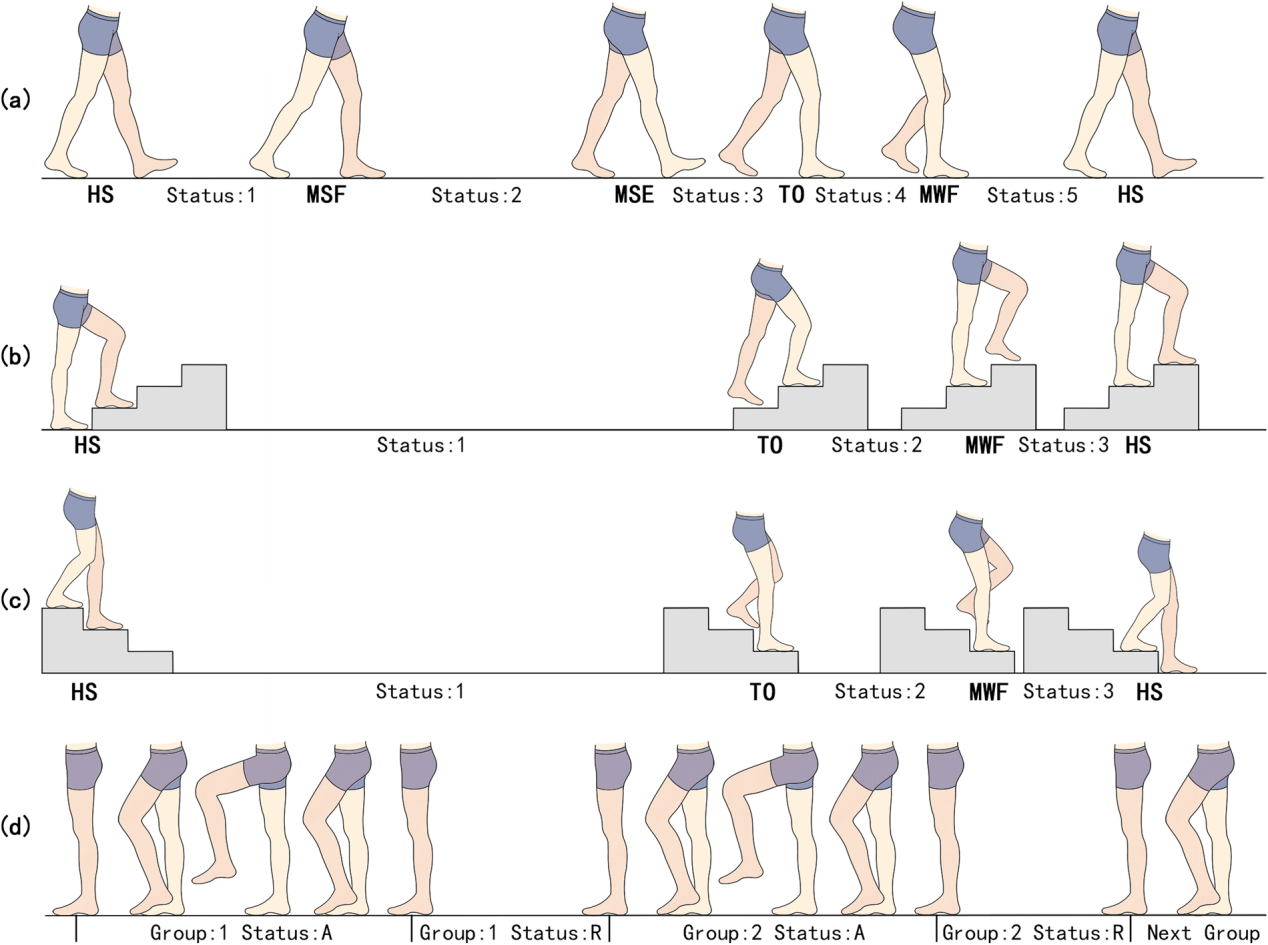
Description of the labels associated with the limb movements. (**a**) Shows the labels for WAK; (**b**) Shows the labels for UPS; (**c**) Present the labels for DNS; and (**d**) Represent the labels of other movements.

The first type of label includes ‘A’ and ‘R’ (Fig. 4), corresponding to the active or rest status of the subject with respect to their lower limb movements. These labels are determined by the force platforms (described in ‘Experimental setting and Equipment’ section) and kinematics data.

The second type of label marks the different gait phases (Fig. 4). Thus, there are five key gait events in the level walking scenario which include heel strike (HS), maximum stance flexion (MSF), maximum stance extension (MSE), toe-off (TO), and maximum swing flexion (MWF)^25–27^, three of which include HS, TO, and MWF, can be detected when going upstairs and downstairs, which were considered in our study. However, since the time from TO to MWF is short during stairs descent (DNS), the entire swing period is counted as the same state. MSF and MSE represent the moments during the stance phase when the knee flexion and extension angles respectively reach their maximum values. MWF refers to the moment of maximum knee flexion during the swing phase. The force platforms and kinematics data were used to detect these events, and then the number of labels were used to distinguish the data between the two consecutive gait events.

## Data Records

**Fig. 5:**
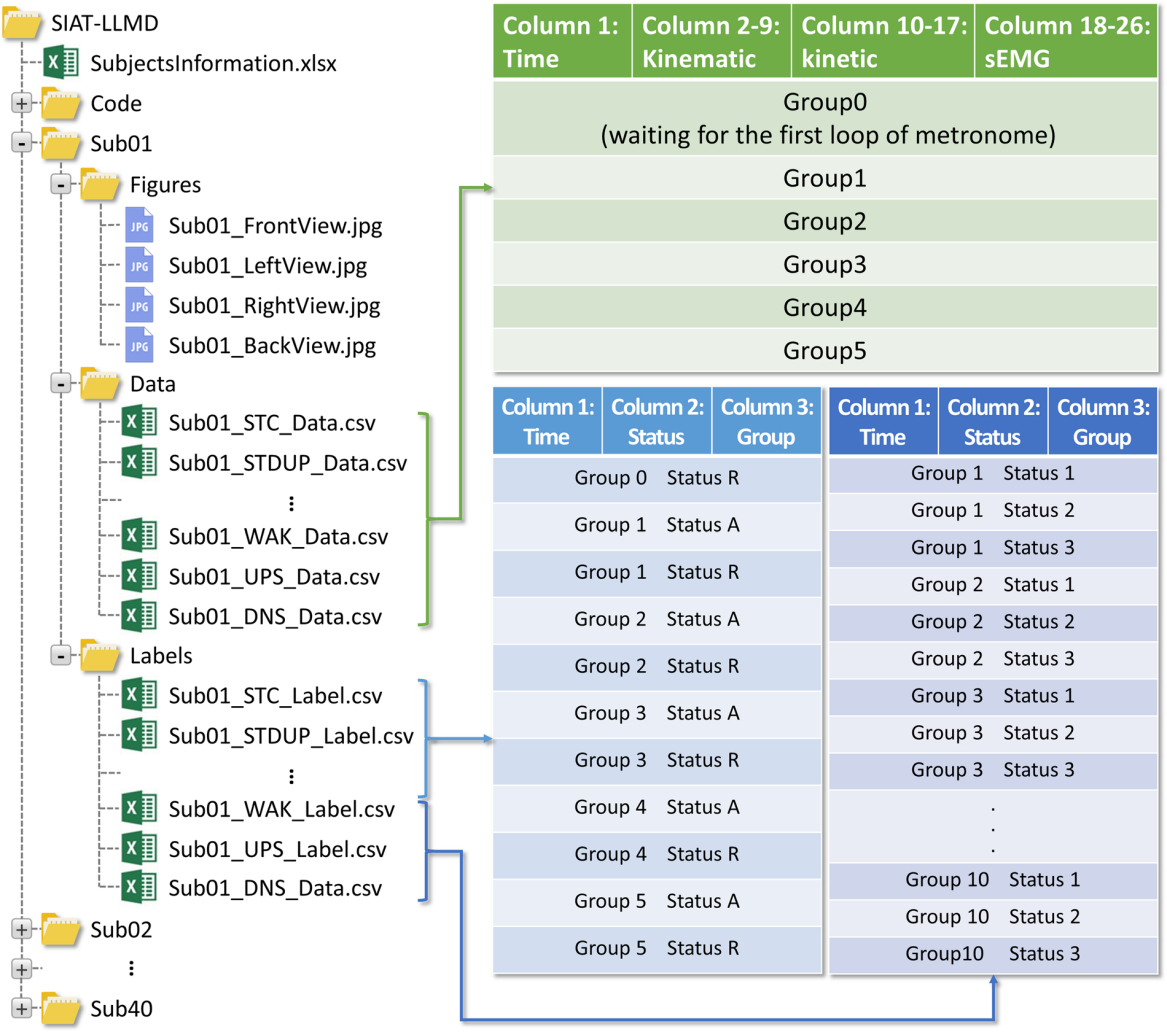
An image indicating how the acquired data and associated information are structured.

The SIAT-LLMD includes the kinematic, kinetic, sEMG dataset, the details of the subjects, photos of experiments, and corresponding labels, which were recorded as shown in Fig. 5. The subjects’ basic information is contained in a file denoted as ‘SubjectsInformation.xlsx’ which holds the subject ID, age, body weight, and body size of each subject. Besides, each subject’s data is organized in a single folder that contains 16 data files with a naming format of ‘Subxx_xxx_data.csv’. In each data file, the first column holds time; kinematic data are recorded in the second to ninth column; the kinetic data are recorded in the tenth to seventeenth column; and the sEMG data are recorded in the eighteenth to twenty-sixth column. In addition, there is a clear header marked with the specific name of each kind of data in the file for easy understanding. The kinematic data from left to right are the joint angle of left hip adduction, left hip flexion, left knee flexion, left ankle flexion, right hip adduction, right hip flexion, right knee flexion, and right ankle flexion, respectively. The kinetic data from left to right are the joint torque of left hip adduction, left hip flexion, left knee flexion, left ankle flexion, right hip adduction, right hip flexion, right knee flexion, and right ankle flexion, respectively. And the sEMG data from left to right are the data collected from the tensor fascia lata, rectus femoris, vastus medialis, semimembranosus, upper tibialis anterior, lower tibialis anterior, lateral gastrocnemius, medial gastrocnemius, and soleus muscles in the left leg, respectively. The corresponding labels of these data are organized in another folder that contains 16 label files with the naming format of ‘Subxx_xxx_Label.csv’.

## Technical Validation

### Repeatability

**Fig. 6:**
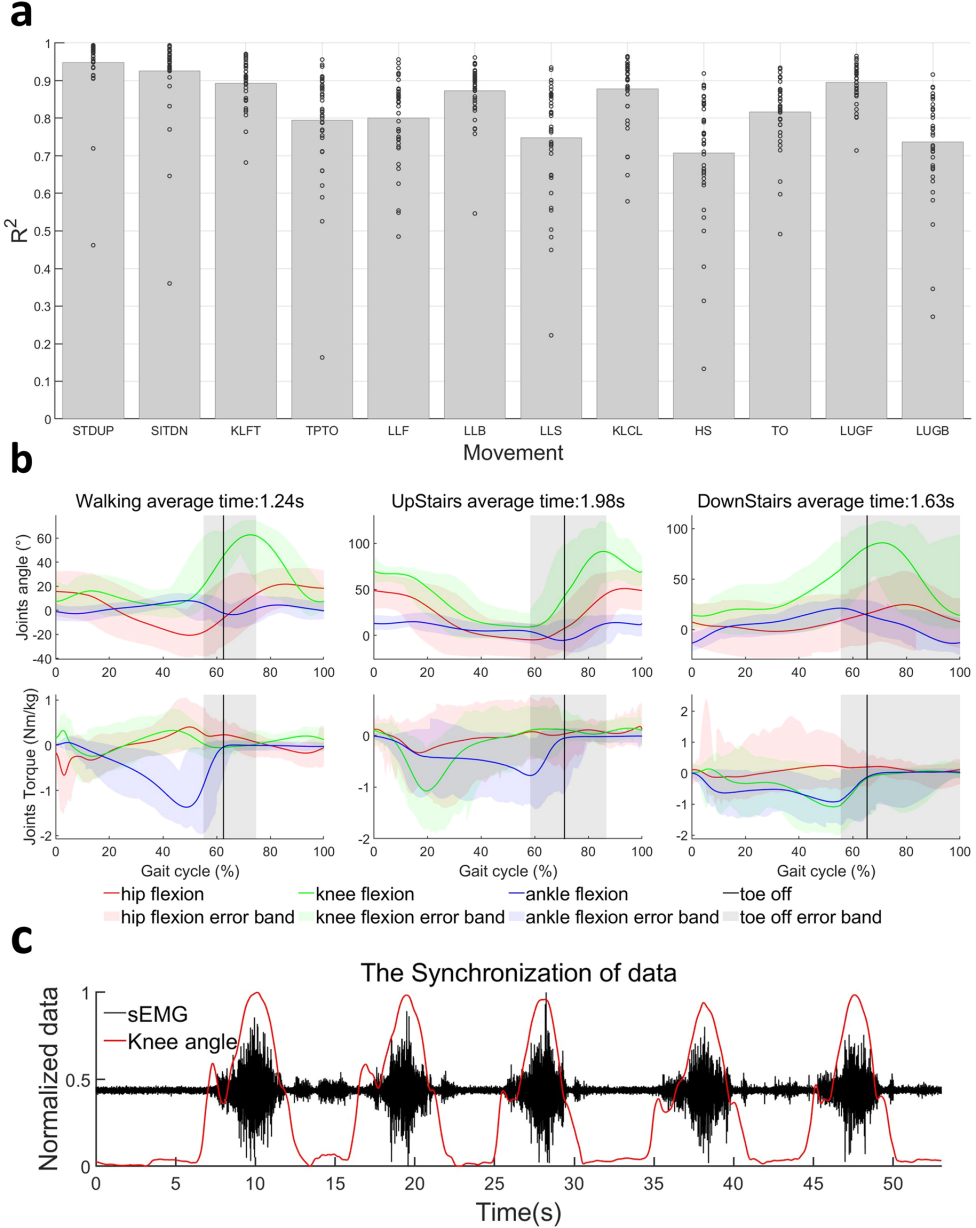
Analyses of the data repeatability and synchronisation. (**a**) The cycles show the average coefficient of determination ($\overline{{R}^{2}}$) between the average angle and the joint angle of each movement for each subject. The bar plots show the average $\overline{{R}^{2}}$ of each movement. (**b**) The average gait cycle of walking on level ground, upstairs, and downstairs. (**c**) The synchronisation between the sEMG acquisition system and motion capture system.

Data repeatability often reflects the consistency and accuracy with which the subjects performed the movements. This phenomenon would normally influence data analysis outcomes in the case of pattern recognition and generalization of the data characteristics. Therefore, it is essential to investigate the data repeatability of the acquired dataset using multiple measures. Thus, the data repeatability has been examined and validated across subjects and evaluations metrics in a stepwise procedure as follows. Firstly, the active processes (STDUP, SITDN, KLFT, TPTO, LLF, LLB, LLS, KLCL, HS, TO, LUGF, and LUGB) of each subject were extracted from their individual data files. Secondly, the average angle of hip adduction, hip flexion, knee flexion, and ankle flexion in the left leg was calculated and stored. Thirdly, the average coefficient of determination between the mean angle and the joint angle was calculated. And finally, the R-square scores of each movement per subject were obtained and the average R-square values of the four joint angles were weighted to arrival at the final value, $\overline{{R}^{2}}$. The weights corresponding to the four joint angles were then calculated according to the relative size of their motion ranges in each movement. This final value, $\overline{{R}^{2}}$ represent a valid means for assessing the data repeatability of each movement per subject. The statistical bar plots (Fig. 6a) show that the $\overline{{R}^{2}}$ values for the twelve movements (excluding WAK, UPS, DNS, and STC), most of them above 0.80, indicating good repeatability. The circles in Fig. 6a show the distribution of ${R}^{2}$ values of 40 subjects for twelve movements.

Moreover, the gait cycle data of WAK, UPS, and DNS were extracted for calculating the average results of hip angle, knee angle, ankle angle, hip torque, knee torque, and ankle torque (as shown in Fig. 6b). Compared with other researchers’ results, the data in this work are most similar to the result of J. Camargo *et al*.^16^. They recorded WAK, UPS, and DNS with different speeds in their dataset, and the WAK data between slow speed and normal speed is closer to our result than fast speed, which reflects the average walking speed in our proposed dataset (SIAT-LLMD). At the same time, we also noticed some differences: our data on the minimum ankle angle is bigger than the results of J. Camargo *et al*.^16^, but smaller than E. Reznick *et al*.^28^. For the UPS, our results are close to the results between fast speed and normal speed in the work of J. Camargo *et al*.^16^ but still have some differences. Our minimum ankle angle in DNS is bigger than their results but similar to E. Reznick *et al*.^28^. Also, our maximum hip angle in DNS is about 7 degrees smaller than J. Camargo *et al*.^16^ and much smaller than the other datasets (the difference between these datasets about this value is also more than 20 degrees)^14,28,29^. The minimum and maximum hip angle in UPS of these datasets are different (the results of SIAT-LLMD are smallest), but the angular range in all these datasets are around 50 degrees^14,16,28,29^. For STDUP and SITDN, the kinematic and kinetic data can be mutually verified with the research of C. Pinheiro *et al*.^30^. However, it should be noted that due to the limited number of steps in the staircase, steady stair ascent or descent may not be achievable, which is a potential limitation of this dataset. Another limitation of this dataset is that the movements were chosen to achieve the above goals as much as possible, but it is important to note that the subjects without any special training may not be able to reach the same levels of performance as highly trained individuals due to limitations in their physical fitness and balance ability.

### Synchronization

During the data collection, the experiment and equipment were set up in a manner that ensured the synchronization of the different data (sEMG recordings and data from the Motion Capture System). Figure 6c demonstrates the normalized knee angle and the normalized sEMG signal along the same time axis, where the angle changes rapidly with the strength of the sEMG signal, indicating the synchronization.

### Classification and regression

To assess the extent to which the various classes of lower limb movements could be decoded, a support vector machine (SVM) classifier (that employs a polynomial Kernel function of order 2, ‘one vs one’ method) and a k-nearest neighbour (KNN) classifier (with K = 5) were applied to classify the 12 movements; a the same SVM and KNN (with K = 1) classifiers were utilized to classify the different gait periods during WAK, UPS, and DNS. In addition, a Gaussian process regression model was employed to predict the angle and torque associated with the joints during WAK, UPS, and DNS. The classification and regression performances were individually evaluated for each subject. Before building the classification and regression models, the sEMG data went through a sequence of filtering operation via filters that includes: notch (with a frequency: 50 Hz; filter quality factor: 100), Butterworth (with low cut-off frequency: 15 Hz; high cut-off frequency: 400 Hz; sampling frequency, fs: 1920 Hz, and order: 7), and wavelet and packet (with wavelet packet threshold: 0.08; wave packet threshold setting: soft; wavelet packet type: ‘db7’; level: 9). Subsequently, the filtered data was segmented using a sliding window (window size: 150 sample points for the classification of 12 movements; window size: 80 sample points for the classification of gait phase) and the Du’s sEMG feature set^31^ (include Integrated EMG, variance of EMG, waveform length, zero crossing, slope sign change, and Willison amplitude) was extracted. It should be noted that the feature set was normalized by using Min-Max scaling normalization method, and subsequently used for the characterization of the movement classes.

**Fig. 7:**
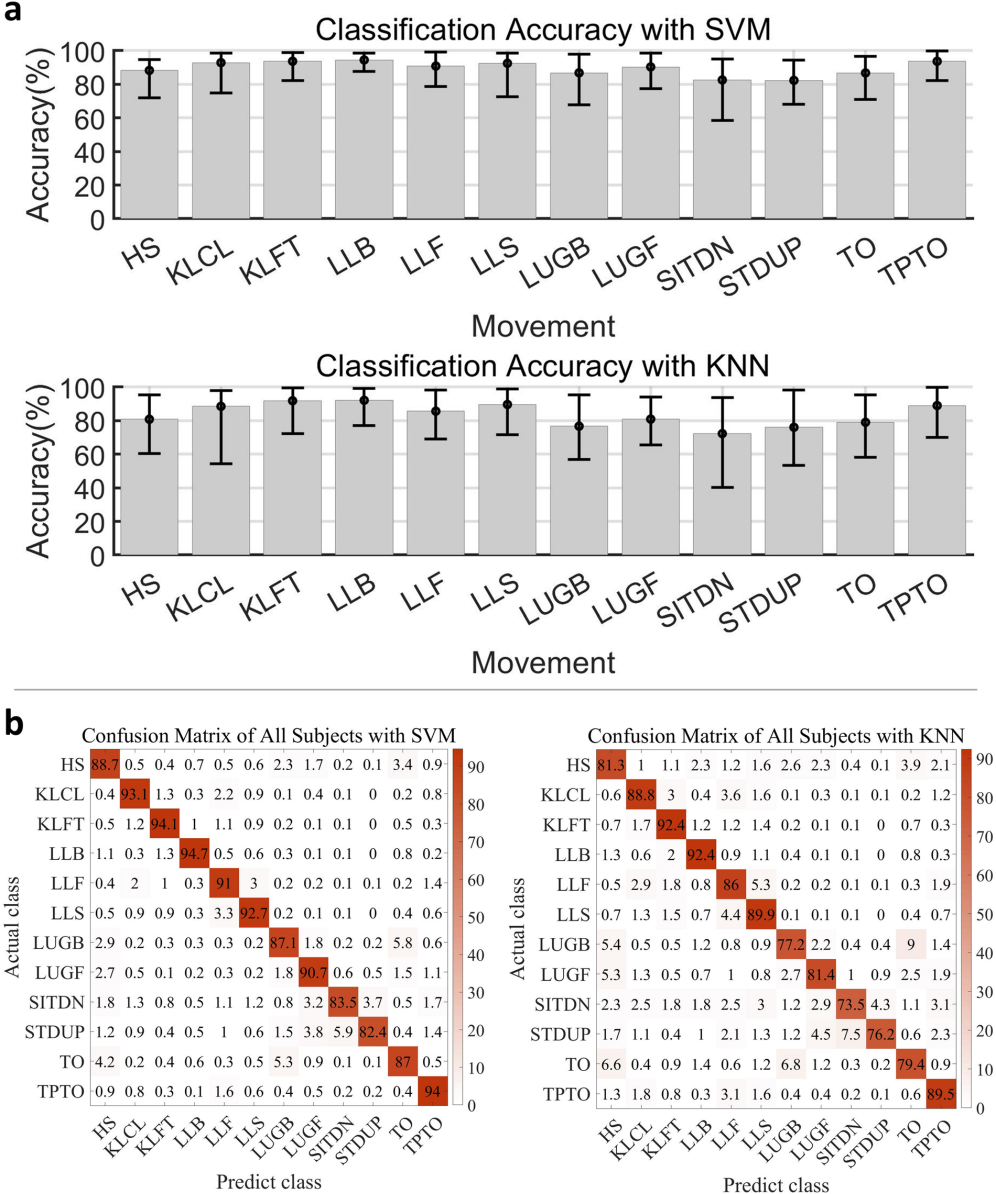
Experimental results of SVM and KNN classification for 12 movements. (**a**) The statistical bar plot shows the average accuracy of each movement. The error bar shows the distribution of accuracy of each movement from the 40 subjects; (**b**) A confusion matrix summarising the results of all subjects.

**Fig. 8:**
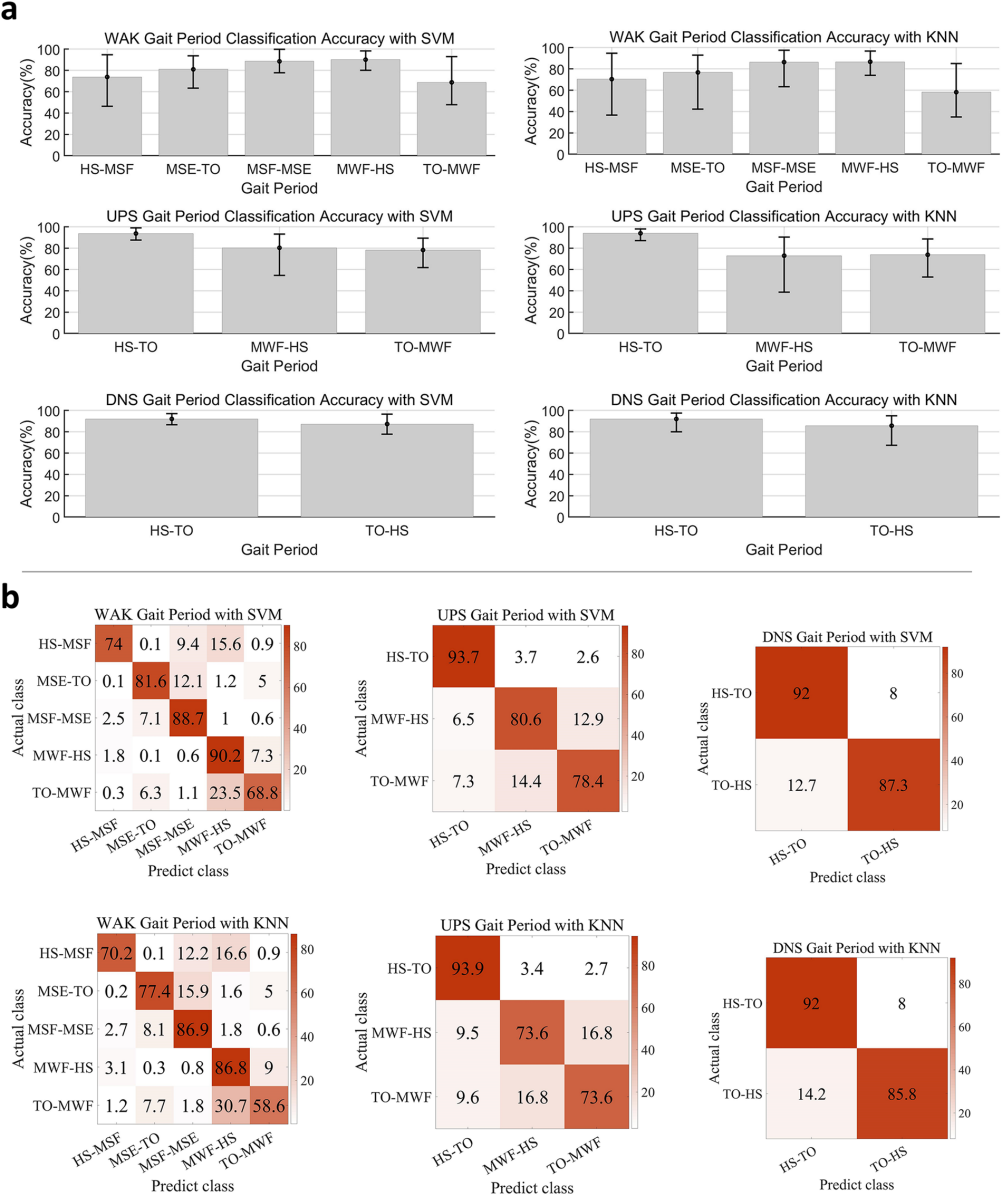
The classification results of gait analysis. (**a**) The statistical bar plots show the average accuracy of each gait phase. The error bars show the distribution of accuracy of each gait phase from the 40 subjects; (**b**) The confusion matrix summarises the results of all subjects.

With the SVM classifier, the classification results show that the average accuracy of the 12 movements is 90.74%, while the WAK gait phase classification achieved an accuracy of 84.50%%, UPS gait phase recorded an accuracy of 88.77%, and DNS gait phase had an accuracy of 90.38%. Meanwhile, the KNN classifier yielded classification results with average accuracy of 85.06%%, across the 12 movement classes while the gait phase average classification results are 80.62% for WAK, 86.82% for UPS, and 89.78% for DNS. The results have been detailed in Figs. 7a, 8a with statistical bar plots indicating that the average accuracy of each movement or gait phase is much above the random selection probability. The error bars (Figs. 7a, 8a) reveal the distribution of the accuracy of each movement from the 40 subjects, presenting acceptable variance. Furthermore, the confusion matrix (Figs. 7b, 8b) shows that the movement with a lower accuracy has other movements similar to it, thereby confirming the reasonability of the results. To train and test our model, we utilized the 5-fold cross-validation technique provided by MATLAB’s Statistics and Machine Learning Toolbox.

**Fig. 9:**
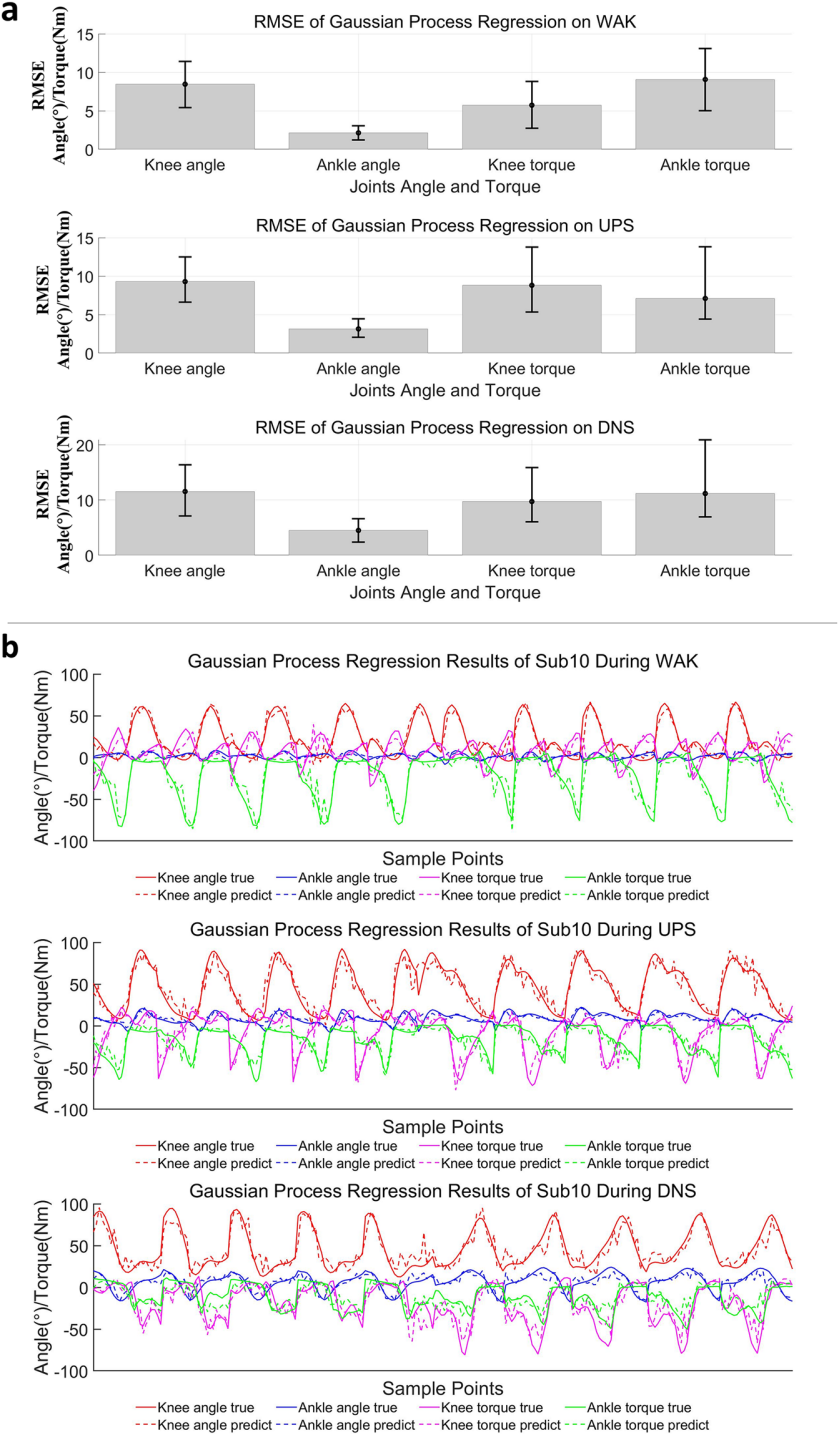
The regression results of gait analysis. (**a**) The statistical bar plot shows the average RMSE of each joint angle and torque. The error bar shows the distribution of RMSE of each joint angle from the 40 subjects; (**b**) The target and predicted curves for a representative subject (Sub10).

For the regression analysis, the statistical bar plots (Fig. 9a) show the average Root Mean Square Error (RMSE) of each joint (the lower, the better), while the error bar (Fig. 9a) represents the distribution of RMSE of each joint-angle and joint-torque from the 40 subjects (the lower, the better). These errors are much smaller than their usual range as reported in a previous study^32^, indicating that the sEMG signal can effectively aid the prediction of the angle and torque associated with the joints. In addition, Fig. 9b shows the regression effect to be more intuitively, with the predicted and actual values presenting the same trend. In conclusion, the above results have reiterated the applicability of our dataset (SIAT-LLMD) from a multi-analysis perspective for lower limb movement intent recognition and gait phase characterization.

## Data Availability

A set of codes for reading, pre-processing of sEMG, splitting of sEMG into windows of various sizes, extracting of sEMG features (including 20 kinds of features^33^ that can be combined into Du’s feature set^31^, Hudgins’s feature set^34^, and novel time-domain feature set^35,36^), normalization of extracted features, generation of sample data, and making log files are provided for easy handling of the data. In addition, some programs in the technical validation section were also included, which can be found from on GITHUB via the following URL: https://github.com/WH-Wei/SIAT-Lower-Limb-Motion-Dataset-Codes.git.
